# Effect of Systemic Infliximab Therapy in Patients with Sjögren’s Syndrome

**DOI:** 10.4274/tjo.48379

**Published:** 2015-08-05

**Authors:** Elif Betül Türkoğlu, Serpil Tuna, Sevil Alan, Mehmet İhsan Arman, Yaşar Tuna, Mustafa Ünal

**Affiliations:** 1 Akdeniz University Faculty of Medicine, Department of Ophthalmology, Antalya, Turkey; 2 Akdeniz University Faculty of Medicine, Department of Physical Therapy and Rehabilitation, Antalya, Turkey; 3 Akdeniz University Faculty of Medicine, Department of Dermatology, Antalya, Turkey; 4 Akdeniz University Faculty of Medicine, Department of Gastroenterology, Antalya, Turkey

**Keywords:** Dry eye, infliximab, Sjogren’s syndrome, Tumor necrosis factor

## Abstract

**Objectives:**

To investigate the effect of systemic infliximab therapy on tear function tests and the ocular surface in patients with Sjögren’s syndrome secondary to various autoimmune diseases.

**Ma­te­ri­als and Met­hods:**

This prospective study included 22 eyes of 22 patients with Sjögren’s syndrome who began treatment with systemic infliximab. Tear film break-up time (TBUT), anesthetized Schirmer’s 1 test, fluorescein staining test, and Ocular Surface Disease Index (OSDI) scores were recorded before treatment and in the 3rd and 6th months of treatment.

**Re­sults:**

In the 3rd month of infliximab therapy, no significant changes were observed in Schirmer’s values, TBUT, fluorescein staining, or OSDI scores (p=0.260, p=0.357, p=0.190 and p=0.07, respectively). In the 6th month of infliximab therapy, no significant changes were observed in TBUT, fluorescein staining, Schirmer’s value or OSDI scores (p=0.510, p=0.320, p=0.220 and p=0.344, respectively).

**Conclusion:**

Infliximab therapy, which is commonly used in systemic autoimmune diseases such as rheumatoid arthritis, Crohn’s disease, ulcerative colitis, and ankylosing spondylitis, did not show a positive effect on ocular surface and tear function tests.

## INTRODUCTION

Sjögren’s syndrome (SS) is a chronic autoimmune disease that affects all the exocrine glands, especially the salivary and lacrimal glands, resulting in mouth and eye dryness.^1^ It may develop secondarily to connective tissue diseases such as primary or rheumatoid arthritis (RA) and systemic lupus erythematosus.^2^ Topical treatment is initially used to manage symptoms; if this insufficient, systemic corticosteroids and immunosuppressive agents are used. In recent years, the tumor necrosis factor alpha (TNF-α) antagonists infliximab and etanercept have been used for this purpose and there are a few studies reporting the results.^3,4^ Zhu et al.^5^ reported increased tear production when TNF-α was inhibited in a rabbit model of autoimmune dacryoadenitis. Li et al.^6^ reported increased tear production and conjunctival goblet cell count and decreased inflammatory cells and cytokines on the ocular surface after topical infliximab treatment in a mouse model of experimental dry eye.

As infliximab has been successfully used in recent years to treat several autoimmune diseases, we aimed in this study to investigate the effects of systemic infliximab treatment on tear function tests and the ocular surface.

## MATERIALS AND METHODS

This prospective study included 22 patients diagnosed with secondary SS according to the American-European Consensus criteria who were going to begin systemic infliximab treatment. After approval by the local ethics committee, detailed information about the study was provided and informed consent was obtained from all subjects. Slit-lamp examination was performed to evaluate anterior segment structures. Patients with eyelid pathologies possibly due to ocular surface changes, patients with a history of ocular surgery or trauma, patients with acute or chronic ocular infections and those receiving topical or systemic corticosteroid therapy for any other reason were excluded from the study. The protocol for systemic infliximab (Remicade; Schering-Plough, Quebec, Canada/Centocor, Malvern, PA) treatment was 5 mg/kg intravenous (IV) for Crohn’s patients and 3 mg/kg IV for other patients, administered in week 0, week 2, week 6 and once every 8 weeks thereafter. Patients continued their artificial tear therapy.

Tear film break-up time (TBUT) test and Schirmer’s 1 test (under local anesthesia) were conducted at the initial examination following routine eye checks, before beginning anti-TNF-α therapy and at 3 and 6 months after beginning medication. At each visit, patients completed the Turkish version of the Ocular Surface Disease Index (OSDI) questionnaire before the tests were conducted. The OSDI has three sections with a total of 12 questions assessing dry eye severity and its impact on visual acuity, daily activities and life quality. In each section, ocular symptoms are given a point value between 0-4 (0=none of the time, 4=all of the time); points corresponding to the patients’ answers are summed and multiplied by 25, then divided by the number of applicable questions, resulting in a OSDI score between 0 and 100.

TBUT was measured without topical anesthetic by wetting a fluorescein strip (BioGlo Sterile Strips, Rose Stone Enterprises, CA, USA) with saline and touching it to the inferior fornix. Patients were instructed to blink three times to distribute the fluorescein. The tear film was examined under ample lighting using a microscope with slit-lamp and cobalt-blue filter and the tear film break-up time was noted. The measurement was repeated several times and the average value was taken. A TBUT of less than 10 seconds was accepted as pathologic.

For the Schirmer’s test, topical anesthetic drops were applied, then standard Schirmer test strips (TearFlo Sterile Strips, Rose Stone Enterprises, CA, USA) were placed at the inferior fornix with one third of the strip under the lower eyelid. After five minutes, the moistened portion of the strip was measured in mm. A result of less than 6 mm was accepted as pathologic.

For the fluorescein ocular surface staining test, one drop of preservative-free artificial tear solution was applied to a fluorescein strip which was touched to the inferior palpebral conjunctiva to dye the tears. The ocular surface was examined by slit-lamp biomicroscopy. According to the staining pattern and detected ocular surface damage, dry eye was categorized by the Oxford grading scheme, in which the conjunctiva and cornea are evaluated together. It consists of five panels (A-E) with anterior segment pictures illustrating degrees of ocular surface damage. Panel A corresponds to grade 0, panel E to grade 4.

All statistical analyses were performed using SPSS version 17.0 (Statistical Package for Social Sciences Inc., Chicago, IL, USA). The patients’ OSDI, TBUT and Schirmer’s 1 values from the three time points were compared using the Student’s t-test. For each patient, values from the eye with more severe symptoms were used in the statistical analysis. Level of significance was accepted as α=0.05.

## RESULTS

The study included 22 eyes of 22 patients who regularly appeared for examinations during the 6-month follow-up period. The patient group had a mean age of 42.9±10.7 years (range, 15-55 years) and consisted of 12 (54.5%) males and 10 (45.5%) females. Infliximab infusion was administered in the Rheumatology and Gastroenterology clinics for RA in 12 patients, Crohn’s disease in 4 patients, ankylosing spondylitis in 3 patients and psoriatic arthritis in 3 patients. The patients’ Schirmer’s values, TBUT, fluorescein staining test results and OSDI scores before beginning treatment and at 3 and 6 months after beginning treatment are summarized in Table 1.

There were no significant differences in Schirmer’s values, TBUT, fluorescein staining or OSDI scores after 3 months of infliximab treatment (Table 1, p=0.260, p=0.357, p=0.190 and p=0.07, respectively). After 6 months of infliximab treatment, there were still no significant differences in TBUT, fluorescein staining, and Schirmer’s or OSDI scores (Table 1, p=0.510, p=0.320, p=0.220 and p=0.344, respectively).

No side effects of the drug other than nausea and diarrhea were observed in our 12 patients during treatment.

## DISCUSSION

In this study we aimed to investigate the effect of systemic infliximab treatment on tear function tests in patients diagnosed with secondary SS. No statistically significant differences in TBUT, fluorescein staining test, OSDI and Schirmer’s scores were observed in patients receiving systemic infliximab treatment.

Findings of inflammatory cell infiltration of the conjunctival epithelium and high levels of immune activator stimulants like human leukocyte antigen (HLA-DR), intercellular adhesion molecule 1 (ICAM-1) and inflammatory cytokines such as interleukin-1 (IL-1), IL-6, IL-8 and TNF-α in SS suggest that the development of dry eye is due to ocular surface inflammation.^7,8,9^ Studies demonstrating that proinflammatory cytokines play a role in the pathogenesis of dry eye have lead to the introduction of anti-inflammatory treatment protocols in the management of this disease.^10,11,12^

The proinflammatory cytokine TNF-α plays an important role in the pathogenesis of many inflammatory diseases by inducing other proinflammatory cytokines and adhesion molecules. In dry eye syndrome, it is also one of the major proinflammatory cytokines found in tears.^6,13^ The TNF-α inhibitor infliximab is a monoclonal antibody that binds both the soluble and transmembrane proinflammatory cytokine forms of TNF-α. It was approved for use in 1999 by the Food and Drug Administration (FDA). Infliximab therapy, which may cause gastrointestinal and hematological side effects, autoimmune diseases, hepatotoxicity and serious infections like tuberculosis, is now widely used in the treatment of RA, Crohn’s disease, ulcerative colitis, ankylosing spondylitis, plaque psoriasis and psoriatic arthritis.^14^ Its ophthalmic use was first reported in 2001 for the treatment of panuveitis and RA-related scleritis.^15,16,17^ Subsequent studies demonstrated the efficacy of systemic TNF-α blockers in the treatment of refractory ocular inflammatory diseases including refractory uveitis, scleritis and peripheral ulcerative keratitis.^14,18,19^ Also, intravenous or subcutaneous infliximab and etanercept have been used to treat SS patients, with conflicting results.^13,20,21^

In our study, 22 patients with SS secondary to other autoimmune diseases were evaluated for 6 months. In these patients, all of whom received systemic infliximab therapy, no significant changes were seen in TBUT, fluorescein staining test, or OSDI and Schirmer’s scores. In a study by Mariette et al.^13^ in which 103 patients received systemic infliximab (5 mg/kg), no improvements were observed in tear function tests during the 22-week follow-up period, although they did see a 30% improvement in a visual analogue scale measuring pain, fatigue and dryness. According to the results of that study, they determined systemic infliximab ineffective in the treatment of SS. In contrast, in a 2001 prospective study Steinfeld et al.^21^ followed 16 patients for 3 months and observed improvements in subjective and objective dryness symptoms. In 2002, Steinfeld et al.^20^ published the 1-year follow-up results for these patients and reported statistically significant decreases in systemic and local symptoms in the 10 primary SS patients that completed follow-up. The results of these studies indicated that systemic infliximab treatment was effective and safe.

Following these conflicting results, Li et al.^6^ evaluated the efficacy of topical anti-TNF-α application in an experimental study. Increased tear production, improvements in ocular surface irregularities, decreased Th1 cells and inflammatory cytokines on the ocular surface, and increased conjunctival goblet cell density were observed in mice treated with topical infliximab (at concentrations of 0.01% and 0.1%). These results suggest that topical infliximab at 0.01% and 0.1% may be effective in treating dry eye.

Limitations of this study are that the patients’ dry eye was not graded and impression cytology and tear osmolarity measurement were not performed. The strengths of this study are that it was prospective, all of the patients regularly attended follow-up appointments, and it draws attention to the ocular surface effects of a systemic drug commonly used today.

In conclusion, systemic infliximab therapy, widely used in the treatment of autoimmune diseases such as RA, Crohn’s disease, ulcerative colitis and ankylosing spondylitis, showed no positive effect on tear function tests. However, considering the results of previous animal experiments, further studies investigating the efficacy of the topical infliximab in SS patients are warranted.

## Figures and Tables

**Table 1:**
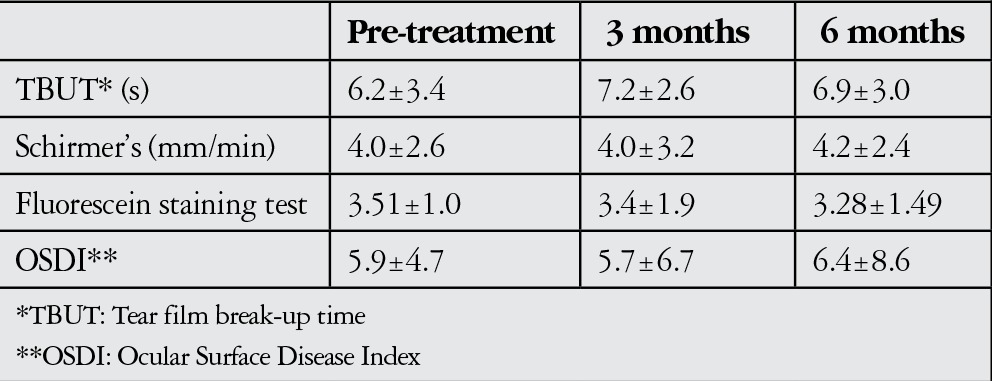
Tear function test results before and during systemic infliximab treatment
